# Repurposed Endoscopes as Accessible Tools for Cadaveric Gastrointestinal Anatomy Instruction in Medical Education

**DOI:** 10.1007/s40670-026-02688-6

**Published:** 2026-03-20

**Authors:** Alicia Nguyen, Clarissa Newman, Manuel Cevallos

**Affiliations:** 1 Department of Medical Education, Creighton University School of Medicine, 3100 N. Central Ave, Phoenix, AZ 85012 USA; 2 Department of Surgery, Creighton University, Phoenix, AZ USA

**Keywords:** Anatomy, Endoscopy, Gastrointestinal, Medical Education, Medical Students

## Abstract

A low-cost, repurposed endoscope was tested as a cadaver-based training tool for medical students. The setup offered realistic visualization of gastrointestinal anatomy and helped improve students’ procedural confidence. This affordable and accessible model demonstrates potential for incorporating early endoscopic training into anatomy curricula, especially in resource-limited settings.

Early exposure to medical and surgical procedures is recommended in medical education; however, medical students often have limited exposure to endoscopic procedures due to the high cost of simulators and restricted access to clinical environments. Endoscopic simulators are usually shared between departments or used in patient-based instruction, both of which are difficult to scale [1]. Additionally, scheduling live demonstrations and coordinating faculty and patient availability further reduce opportunities for hands-on training. Delays in gaining procedural familiarity and decreased student confidence in anatomy-based skills have been observed [1–3]. Therefore, cadaver-based endoscopy can help close this educational gap by providing anatomically accurate, hands-on experience in the anatomy lab setting [3].

To address this, we developed and piloted a low-cost, repurposed borescope for cadaver-based gastrointestinal (GI) training. The new “endoscope” equipment includes: (A) a TD 450 S dual-lens borescope with LED illumination, a 4.5-inch display, 1080p resolution, and a 2–10 cm depth of field; (B) an insufflator: a manual air pump to maintain pressure at 6–10 mmHg; and (C) standard lab tools, such as hemostats and scalpels, used to create entry points of 0.5–1 cm for scope insertion. Students collaborated to explore GI structures using a formaldehyde-preserved cadaver from the anatomy lab at Creighton University – Phoenix. This simple, affordable setup demonstrates that meaningful procedural learning can happen outside traditional simulation environments with basic, low-cost tools. The new “endoscope” was showcased at the American Association of Clinical Anatomists’ annual meeting in Seattle, WA, in June 2025.

This initiative highlights a resourceful adaptation transforming anatomy education. By repurposing a consumer-grade borescope costing less than $100, we developed a training model that provides realistic visualization and hands-on experience without financial burden. The borescope setup enabled clear viewing of internal anatomical structures and gave students the chance to practice essential endoscopic techniques. Key landmarks, such as stomach rugae, the pyloric canal, and duodenal folds or plicae circulares, were identified. Post-procedure feedback showed increased confidence in scope handling and anatomical orientation, along with greater familiarity with endoscopic navigation. Qualitative feedback emphasized teamwork and the novelty of applying anatomy knowledge in a real procedural setting. Although the scope has limitations in articulation and the color variation of cadaver tissue, the borescope remains an educational, engaging, and practical tool for procedural training (Fig. 1).

**Fig. 1:**
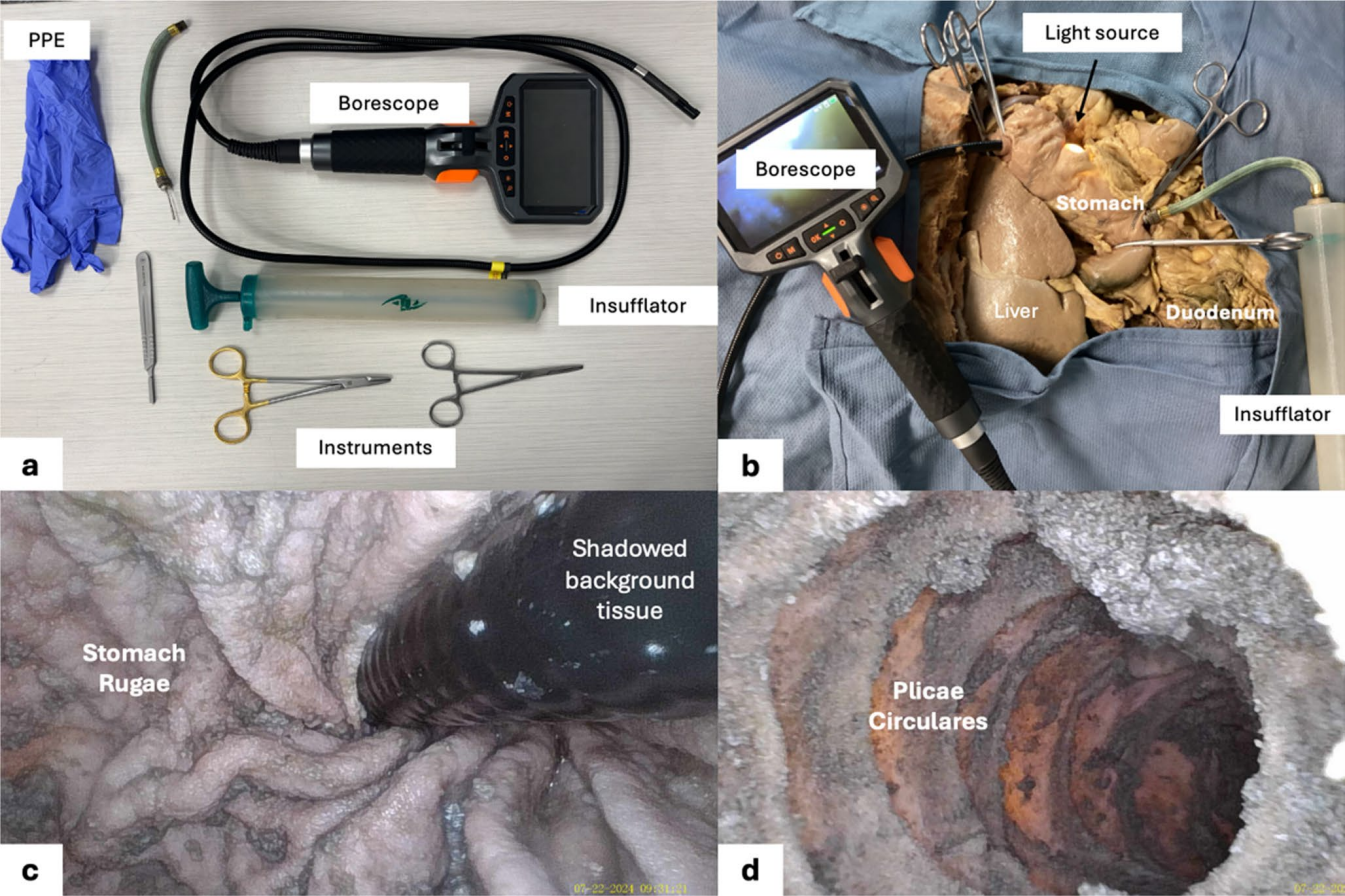
(**a**) Equipment used for cadaveric gastrointestinal visualization, including a repurposed borescope and a manual air insufflator (hand pump), along with standard anatomy laboratory instruments and personal protective equipment. (**b**) Setup during cadaveric exploration demonstrating borescope placement, external light source, and insufflation to visualize the stomach and proximal duodenum. (**c**) Representative endoscopic image obtained using a repurposed borescope, demonstrating gastrointestinal mucosal folds (gastric rugae) with adjacent shadowed background tissue visible. (**d**) Endoscopic view of plicae circulares within the proximal small intestine.

This innovation highlights the importance of accessibility and creativity in medical education. When cost barriers are lowered, institutions can provide more students with procedural experience, ensuring that all students, regardless of their resources, develop a sense of curiosity and confidence in procedures early in their training.

Anatomical limitations: Donors were preserved in formaldehyde. This method causes tissue stiffness, which makes procedures like oral endoscopy intubation and passing through the esophageal and pyloric sphincters more difficult. To overcome these obstacles, a small incision was made to facilitate endoscope insertion. Additionally, we recommend clamping the distal part of the organ during evaluation to maintain consistent air pressure and expand the viscera.

Furthermore, anatomy provides a natural environment for integrating procedural skills into the preclinical curriculum. Cadavers offer realistic anatomy in a setting that promotes safety, respect, and teamwork—principles essential to clinical competence. By introducing endoscopic techniques early, we establish a direct link between anatomy and patient care. Although the initial procedure was small in scale, the results indicate broader educational benefits. Using low-cost, repurposed tools can enhance traditional teaching by fostering procedural thinking before students enter clinical settings. As a result, anatomy labs can be transformed into spaces for practical procedural learning with minimal investment, helping students bridge the gap between basic and clinical sciences.

Future directions involve expanding participation to larger groups, standardizing assessment tools, and assessing long-term impacts on procedural skills. Incorporating feedback from structures could further improve learning results. This model’s educational value depends on its accessibility and capacity to encourage peer-based learning in a safe, low-pressure setting.

In summary, using a repurposed endoscope for cadaver-based training shows that innovation in medical education isn’t limited to cutting-edge technology. This method offers an affordable way to connect theoretical anatomy with clinical practice, promoting early procedural skills and teamwork. As medical education continues to advance, adopting a low-cost, high-impact solution can make training more inclusive and practice-focused, ensuring that procedural competence starts in the anatomy lab, not the operating room.

## References

[1] Hassan I, Shiwani MH. Limitations of commercially available endoscopic simulators in endoscopic training. Simul Healthc. 2012;7(3):155–60. 10.1097/SIH.0b013e31823b09f1.22495386

[2] Sun L, Lin Z, Zhang Z, et al. Basic surgical skills can be educated with acceptable efficiency and student satisfaction using distance teaching and homemade tools. Sustainability. 2022;14(14):8639. 10.3390/su14148639.

[3] Balekuduru AB, Dutta AK, Subbaraj SB. Endoscopy on a human cadaver: a feasibility study as a training tool. J Dig Endosc. 2018;9:103–8. 10.4103/jde.JDE_13_18.

